# Threading Precision: Progress and Emerging Trends in Aptamer-Based Nanopore Sensing

**DOI:** 10.3390/bios16080401

**Published:** 2026-07-23

**Authors:** Arghya Sett

**Affiliations:** Independent Researcher, L-4653 Differdange, Luxembourg; arghya.sett@gmail.com

**Keywords:** aptamer, nanopore, single-molecule sensing technology

## Abstract

In recent years, nanopore technology has enhanced analyte detection, enabled higher resolution and achieved single-molecule sensing capability. Aptamer-conjugated nanopore sensing technology combines the high specificity of aptamers with the single-molecule resolution of nanopores. By anchoring aptamers to biological, solid-state or hybrid nanopores, target binding events produce distinct electrical signatures that allow sensitive and label-free detection. This approach enables real-time monitoring of small molecules, proteins, and even pathogens, with promising applications in diagnostics, drug screening, environmental monitoring, etc. Hybrid biological/solid state devices produce robust signals and are suitable for PoC applications. The aptamers “magic bullets” have also been exploited to develop single-molecule antigen detection using nanopores, which offers a promising alternative for accurate virus testing to contain their transmission. Chemical conjugation of aptamers to nanopore interfaces improves selectivity for peptides/amino acids and expands robustness for practical samples. Aptamer-based nanopipettes offer high analytical precision by enabling label-free, real-time detection of target molecules in ultra-small sample volumes. This review maps aptamer–nanopore integration across biological, solid-state, and hybrid platforms. It also explores various types of aptamers integrated into nanopore platforms that cater to precise, single-molecule recognition, paving the way for highly sensitive, portable diagnostics and next-generation therapeutic monitoring tools.

## 1. Introduction

Aptamers are short, single-stranded oligonucleotides—either DNA or RNA—that fold into unique three-dimensional conformations, allowing them to bind selectively to specific molecular targets ranging from small ions and metabolites to large proteins and even whole cells. Their remarkable affinity and specificity, comparable to that of antibodies, stem from their structural adaptability and the fine-tuning possible through in vitro selection procedures such as SELEX (Systematic Evolution of Ligands by Exponential Enrichment). Due to their thermal stability, chemical versatility, and reusability, aptamers have gained interest as biorecognition elements in analytical, diagnostic, and therapeutic applications [1,2,3].

Nanopores, on the other hand, represent a breakthrough in single-molecule detection technology. These nanometer-scale orifices—either biological (such as α-hemolysin) or solid-state (such as silicon nitride or graphene)—enable the direct, label-free detection of analytes via modulation of ionic current when molecules traverse or interact within the pore [4,5]. This approach allows real-time analysis with extraordinary sensitivity, often down to the level of individual molecules. The precise control of pore geometry, surface chemistry, and applied potentials has further expanded nanopore platforms into powerful tools for sequencing, biomolecular detection, and high-throughput molecular diagnostics [6].

When an analyte such as DNA, RNA, protein, or small molecule interacts with the nanopore, it temporarily disrupts the ionic current, producing a unique electrical signature that reflects its size, charge, and conformation. The integration of aptamers with nanopore technology represents a promising frontier in biosensing, merging the molecular recognition precision of aptamers with the high-resolution electrical readout of nanopores. In recent years, this hybrid platform has evolved into a versatile sensor system capable of detecting diverse analytes with single-molecule sensitivity and rapid, multiplexed signal output. Yet, despite these advances, challenges remain in nanopore surface functionalization, signal standardization, and reproducibility under complex biological conditions [7]. This review systematically examines recent advances, emerging trends, and various integrative design strategies underpinning the next generation of aptamer-enabled nanopore sensors, with emphasis on their transformative potential in rapid diagnostics, environmental surveillance, and molecular bioanalytical applications.

## 2. Tuning of Aptamer Selection for Nanopore Applications

There are certain improvements and modifications required for SELEX to be compatible with nanopore applications. Certain specific favorable sequences that retain a unique folding structure and binding efficiency under specific conditions (high salt, electric field and confined geometry) are the ideal candidates for nanopore applications. Nanopore-assisted selection consists of a nanopore readout (single-molecule current) as the partitioning/selection signal—which helps to collect sequences that exhibit target-dependent signature sequences. This method can directly enrich the sequences that are “favorable” in nanopore sensors [8,9].

High-throughput SELEX makes it possible to deliberately select aptamers that are not only high-affinity binders but also better suited to the physical and functional constraints of nanopore sensing. By coupling massively parallel sequencing and advanced analytics with modified selection conditions, the process can be tuned so that aptamers remain structurally stable and discriminate under the high-ionic-strength, voltage-driven environments typical for nanopores [10]. High-throughput SELEX allows very large libraries to be screened and monitored across selection rounds, dramatically increasing the chance of discovering aptamers that retain function when confined, tethered, or partially threaded through a nanopore. Deep sequencing of intermediate pools reveals sequence families and structural motifs that persist under stringent conditions, helping to prioritize candidates with robust folding and target recognition compatible with nanopore assays.

Because selection rounds can be run directly under nanopore-like buffer, voltage, and crowding conditions, high-throughput formats support “environment-matched” evolution of aptamers. This means ionic strength, pH, and co-factors can be adjusted to mimic nanopore operation, enriching sequences that maintain affinity and structural integrity during translocation or surface immobilization [11]. Artificial intelligence (AI) has recently emerged as a transformative and effective tool in designing aptamers that are structurally and functionally optimized for nanopore sensing environments. Traditional SELEX often fails to yield specific sequences that remain stable or exhibit clear signal transitions under the high ionic strengths and electric fields typical of nanopore operation. AI-driven methods overcome this limitation by integrating computational modeling, sequence–structure prediction, and data-driven screening tailored to nanopore conditions.

## 3. Strategies of Aptamer–Nanopore Integration

Nanopore sensing relies primarily on measuring ionic current as molecules pass through or interact with a nanoscale pore (biological or solid-state). Aptamers (short, structured nucleic acids) can be integrated into nanopore systems to confer specificity for a plethora of analytes: small molecules, proteins, peptides, etc. There are different types of pf aptamer integration processes with nanopore technology.

### 3.1. Biological Nanopores

α-hemolysin (α-HL) is a widely used biological nanopore. It forms a stable channel in a lipid bilayer, with a well-defined vestibule and constriction region [5,9]. In a recent study Chatterjee et al. described structural insights into nanopore formation of *S. aureus* alpha- hemolysin [12]. Alpha-hemolysin assembles on lipid membranes via metastable pre-pore intermediates before forming the final transmembrane β-barrel pore. These pre-pore states involve oligomerization on the membrane surface, with partial conformational rearrangements but without full membrane insertion. Structural analyses reveal that lipid interactions stabilize intermediate conformations, guiding the transition from soluble monomers to membrane-inserted pores. The transition to the pore state includes large-scale β-barrel formation and membrane penetration, driven by cooperative conformational changes. Understanding these intermediates provides crucial insight into membrane disruption mechanisms and can inform strategies to inhibit toxin activity or engineer nanopore systems.

MspA nanopore (derived from Mycobacterium) has a narrow constriction and has been used for detecting aptamer conformational changes [13]. Aptamers are non-covalently docked into the MspA nanopore (which has a wide vestibule capable of hosting various nucleic acid structures) via a crucial R118 cationic ring, which stabilizes the aptamer inside the pore. The cognate ligands interact from the trans side, bind the aptamer inside the pore and change the ion current signatures, enabling the structural state discrimination. This sensing technology has been validated with three well-known aptamers (dopamine DNA aptamer, serotonin DNA aptamer and theophylline RNA riboswitch aptamer). Upon ligand binding, the aptamer adopts a single stable conformation, causing reduced current fluctuations. This also showed compatibility with both DNA and RNA aptamers and platforms generalizable to riboswitches, engineered regulatory nucleic acid motifs, etc. The advantages of this system include label-free detection, single-molecule resolution and the ability to study dynamic conformational pathways, not only binding endpoints. Currently, the limitations are the sensitivity; the LOD is around 100 nM for dopamine, which is insufficient to detect physiological neurotransmitter levels (1–10 nM). In recent future, with the help of pore charge engineering and aptamer sequence engineering, the sensitivity and efficiency of this sensor can be improved. In another study researchers developed aptamer-functionalized nanopipettes as a label-free platform for detecting viral fragments using ion current rectification (ICR) signals [14]. Specific binding between aptamers and viral targets modulates surface charge inside the nanopipette, producing measurable changes in ionic current. The method also showed high sensitivity and selectivity, with potential for single-molecule-level detection in confined nanoscale environments. This approach offers a promising route toward rapid, low-volume, and portable viral diagnostics, though challenges like biofouling and stability remain.

Researchers have also developed various nanopore-based single-molecule protein sensing approaches, which have various advantages over traditional methods, which suffer from low throughput, limited read lengths, and poor dynamic range [15].

### 3.2. Various Modes of Aptamer Integration in Biological Nanopores

Covalent anchoring of aptamer

In early work, a DNA aptamer (e.g., thrombin-binding aptamer) is hybridized to an oligonucleotide that is covalently attached to a cysteine engineered on the α-HL pore [16,17]. This study presents a new aptamer-based nanopore sensing strategy using the protein nanopore α-hemolysin (αHL). Unlike earlier methods where the sensing ligand is chemically fused to the pore, this work introduces a modular system, where an aptamer is attached indirectly via a DNA adapter tethered to the pore through a disulfide bond. This creates a “reporter” that resides near the pore entrance: the aptamer binding event modulates the ionic current, giving a specific blockade signature.

Host–guest/displacement strategies

In a study, researchers have developed a universal nanopore sensing strategy that integrates aptamer–probe hybridization with host–guest supramolecular chemistry inside the α-hemolysin (αHL) nanopore. The authors engineer a DNA probe modified with a ferrocene–cucurbit[7]uril (Fc–CB[7]) host–guest complex, which produces unique multilevel ionic current signatures when translocating the αHL pore. In the presence of a target analyte (protein or small molecule), the aptamer binds the analyte and releases the modified DNA probe, allowing its translocation and generating highly specific electrical signals. This serves as a quantitative readout, with event frequency correlating with analyte concentration.

The platform is demonstrated for VEGF121, thrombin, cocaine, and MMP9, showing nano- to picomolar sensitivity. The detection limit for VEGF was in the range of 0.1–140 nM with a LOD of 0.1 nM, whereas MMP-9 was quantified from 0.01 to 90 nM with a LOD as sensitive as 10 pM. In the conventional ELISA method, 0.36–4.5 nM of MMP9 can only be detected in clinical samples. Furthermore, the use of magnetic beads (MBs) significantly improves sensitivity by removing excess aptamer–probe duplexes, achieving picomolar detection limits. This universal platform is capable of single-molecule sensitivity and can be upgraded to high modularity, enabling multi-target detection in one sample.

A thrombin-binding 15-mer G-quadruplex aptamer is hybridized to a short oligo linked to the pore opening. When thrombin binds the aptamer, it causes distinct ionic current changes detectable at the single-molecule level. This enables the real-time measurement of thrombin concentration and determination of its kinetic parameters (kon, koff) and dissociation constant (Kd), in agreement with the current established methods.

The study further showed how linker length and aptamer positioning influence pore entry, signal characteristics, and detection efficiency. The platform also allowed convenient switching of aptamer sequences and suggested a path toward parallel nanopore sensor arrays for multiple analytes.

Different magnetic beads also improve sensitivity by concentrating on the released probes, reducing the detection limit by orders of magnitude. Magnetic beads can also help with pre-concentration of analytes. These beads (MBs) can collect target-binding events from larger sample volumes and complex matrices (serum, plasma, etc.). Removing excess aptamer-probe duplexes can eliminate signal interference. Isolating only released probe-DNA generates clean nanopore events. Different aptamers can be immobilized on different bead batches, enabling them for multiple target-sensing. These magnetic beads are functionalized with reporter DNA, which is released after enzymatic cleavage, binding-induced conformational change and competitive displacement. Various applications of MBs include CRISPR nanopore assays (Cas12 cleavage releases DNA from beads), enzyme activity assays, DNA methylation detection, etc.

### 3.3. Integration of Aptamers in Solid State Nanopores

Integration of Aptamers into Solid-State Nanopores:

Solid-state nanopores (e.g., silicon nitride, glass) provide mechanical robustness, tunable geometry, and chemical functionalizability. Aptamers can be grafted onto their inner surface to design a precise, selective binding region for their targets.

Some examples of solid-state nanopores are as follows:

- Researchers have developed a label-free single-molecule sensing platform using aptamer-functionalized glass nanopores to monitor the interaction between carcinoembryonic antigen (CEA)—an important cancer biomarker—and its specific DNA aptamer [17].

A quartz nanopore was first coated with a gold film, enabling functionalization through Au–thiol chemistry. A thiolated CEA aptamer was immobilized on the inner wall of the nanopore, creating a confined environment where CEA molecules could interact with the aptamer during electrophoretic translocation.

Two distinct types of current signatures were observed:

Type I: Fast, shallow blockade events from CEA moving through a bare gold nanopore (no aptamer interaction).

Type II: Deep, long-duration blockades in the aptamer-modified nanopore, indicating binding and dissociation interactions.

By statistically analyzing these single-molecule events, the study extracted association (k_on_), dissociation (k_off_), and dissociation constant (Kd) values at the single-molecule level. The system showed high selectivity, distinguishing CEA from non-target proteins (IgG, BSA, and HRP), and successfully detected CEA in human serum samples, with the results comparable to ELISA [18].

In another study, scientists have tried to integrate amino-acid-specific DNA aptamers into interface nanopores (solid-state) to detect peptides with specific residues. They have developed a force-controlled interface nanopore (iNP) system that integrates DNA aptamers to enable amino-acid-specific detection of peptides. The iNP system uses a microchanneled atomic force microscope cantilever to form a nanopore at the interface with a soft PDMS substrate. This allows for dynamic tuning of the pore size, which leverages unique flexibility and wide applications.

For instance, a phenylalanine aptamer was used, where the aptamer is built into the nanopore interface; its binding to phenylalanine moieties in peptides slows down translocation of those peptides [19]. By tuning the applied voltage (i.e., reducing the voltage), they succeeded in prolonging the aptamer–target interaction time, giving discrete ionic current levels with repeated motifs. Importantly, the system showed high selectivity: peptides containing phenylalanine generate different current signatures than peptides containing structurally similar residues (tyrosine, tryptophan). They also decoupled specific binding (aptamer–target) from nonspecific interactions (electrostatic/hydrophobic) using optical waveguide light-mode spectroscopy. This represents a step toward single-molecule proteomics, since it allows recognition of specific amino acids within peptides.

### 3.4. Hybrid Nanopores

**Figure 1:**
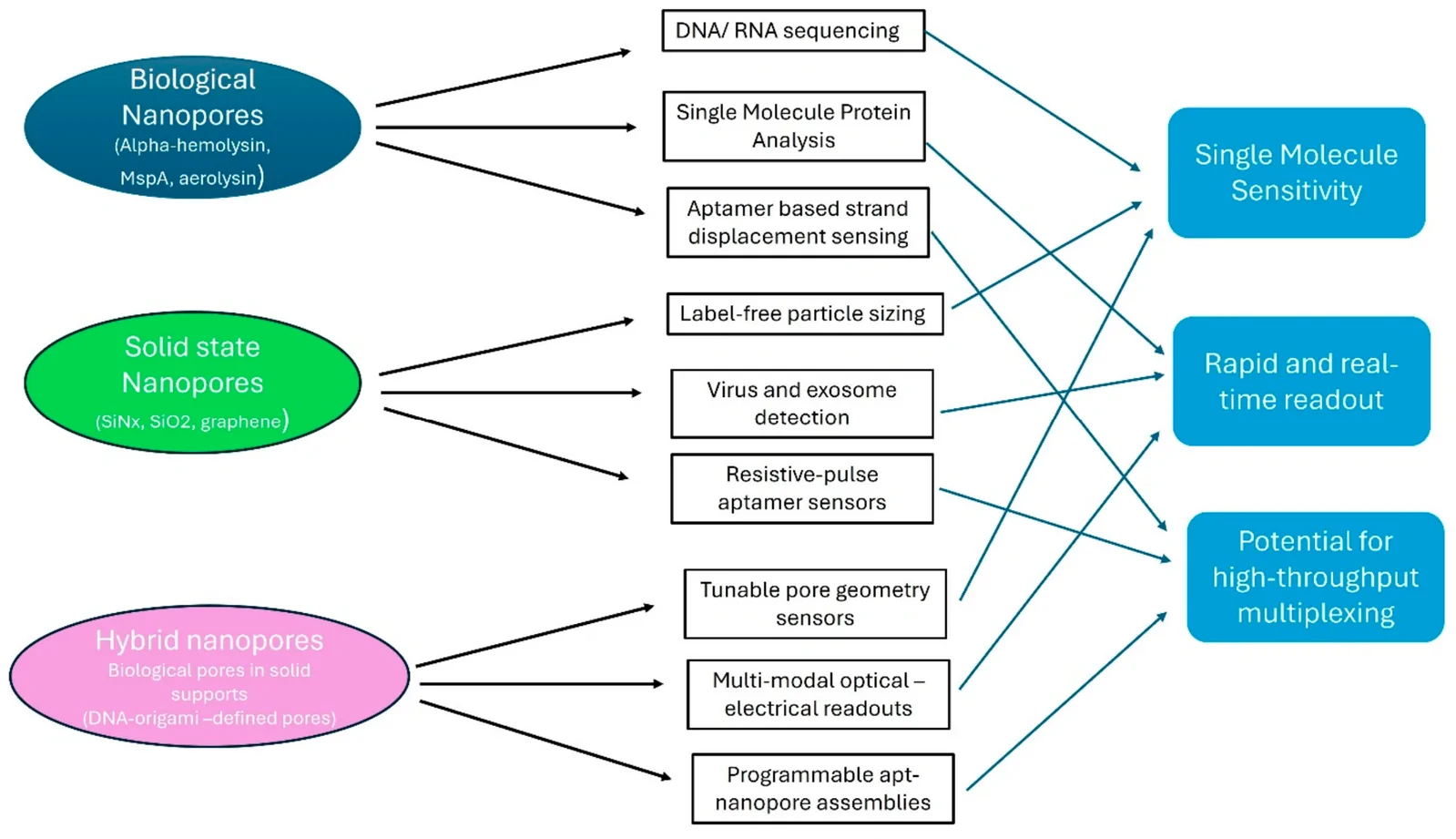
Different types of Nanopores and their versatile applications in aptamer sensing. It illustrates biological (α-HL, aerolysin), solid-state (SiNx, graphene), and hybrid nanopores, each enabling distinct aptasensor capabilities: single-molecule kinetics (biological), robust field deployment (solid-state), and combined precision/stability (hybrids).

Hybrid aptamer–nanopore sensors combine the molecular recognition power of aptamers with the high-throughput signal readout of nanopores. In these systems, biological nanopores provide precise single-molecule discrimination, while solid-state nanopores add mechanical robustness, tunable pore size, and easier device integration. The aptamer serves as a selective bioreceptor, changing conformation or translocation behavior upon target binding and generating a measurable ionic current signal. By integrating both nanopore types, these platforms aim to improve sensitivity, stability, and practical usability for detecting proteins, nucleic acids, and small molecules (Figure 1). Recently, the researchers have developed a nanopipette–DNA origami nanopore hybrid with an ATP aptamer on the central aperture of the DNA origami nanopore, trapped by electrophoresis at the glass nanopipette tip for small-molecule sensing [20].

### 3.5. Mechanistic Insights of Aptamer and Nanopore–Synergistic Insights

Aptamers (magic bullets) fold into highly specific 3D conformations or structures that bind to their cognate targets (small molecules, proteins, cells, etc.), and nanopores detect changes in ionic currents as single molecules translocate or dock through them. Together aptamers act as “molecular locks” and nanopores act as “electrical readers”. The aptamer–nanopore pairing transforms binding events into detectable electrical signals.

**Figure 2:**
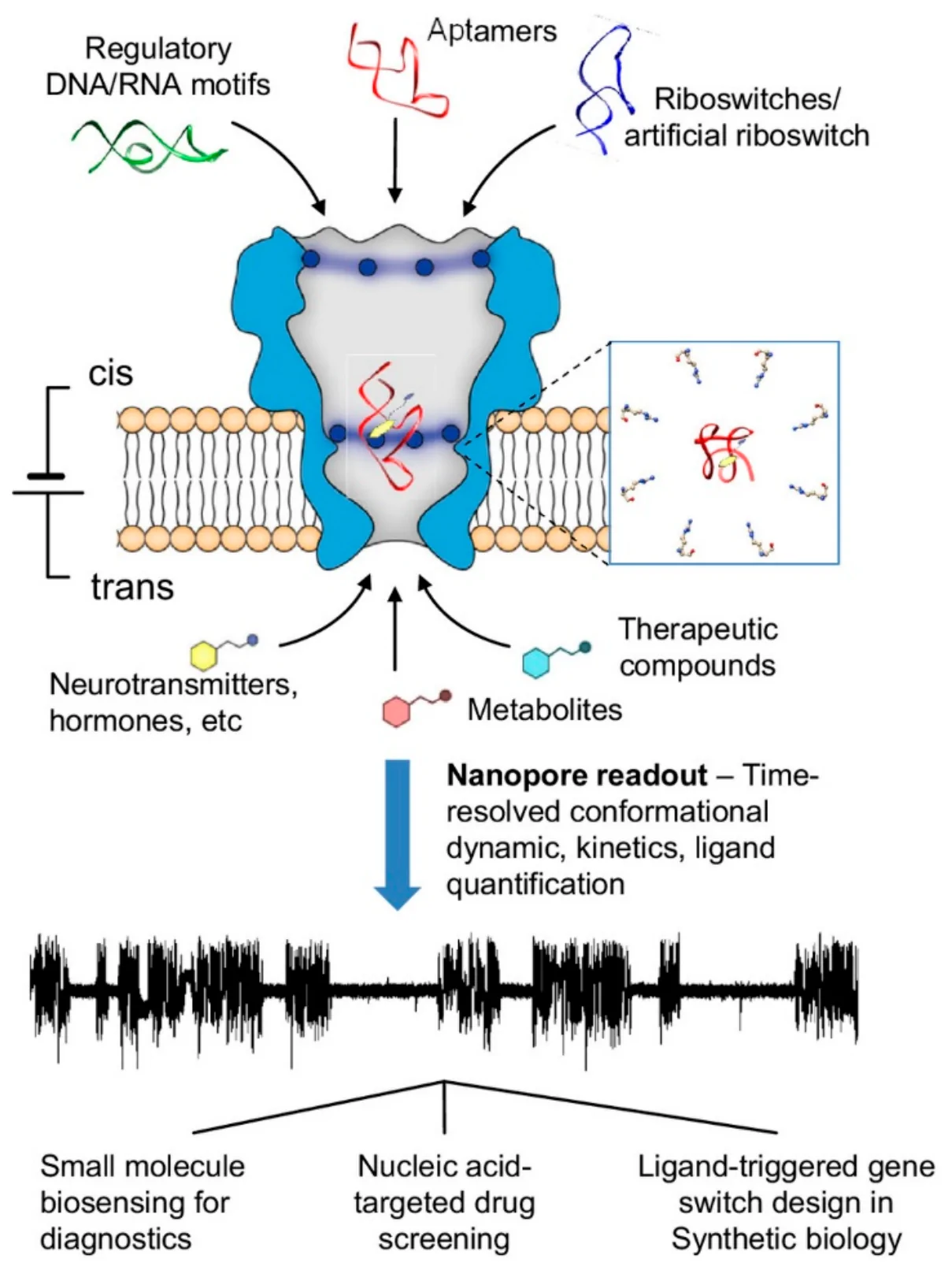
Fundamental concepts and uses of a nanopore platform designed to detect nucleic acid structural shifts upon interaction with small molecules. (Reprinted with permission from ref. [13].)

- Conformational readout: Aptamers often change shape (folding, unfolding) upon binding. When docked or captured in a nanopore, these conformational changes modulate ion current in characteristic ways, which can be resolved at single-molecule timescales (Figure 2). In a study by Chingarande et al., they have developed a real-time label-free detection platform that uses the MspA protein nanopore to noncovalently dock nucleic acid aptamers and monitor their conformational transitions in real-time upon binding to small molecules like dopamine, serotonin, and theophylline [13].
- Kinetic measurements: In the same study, with the help of analyzing the characteristic nanopore current signatures, the platform can quantify ligand binding kinetics, identify key aptamer motifs involved in ligand binding, and demonstrate the selectivity of aptamers for their target ligands [13]. Due to this fact, each binding or unbinding event can be read out as a separate current trace; one can compute association and dissociation rate constants from dwell times.

In another study, a DNA oligonucleotide is covalently attached to the αHL nanopore, acting as an adapter to which various aptamers can be coupled by hybridization. When the thrombin-binding aptamer is hybridized to the adapter, two current blockade levels (B1 and B2) are observed, corresponding to the insertion of the aptamer’s quadruplex domain and the double-stranded DNA segment, respectively. The binding of thrombin to the aptamer results in a new current level (UB + T), allowing the detection and quantification of nanomolar concentrations of thrombin. The approach provides association/dissociation rate constants and equilibrium dissociation constants for the thrombin-aptamer interaction. The rate constants for the aptamer and thrombin interactions were determined by observing the transitions between the UB and UB+T current levels at different thrombin concentrations. The association rate constant Kon and dissociation rate constant Koff were used to determine the equilibrium dissociation constants (Kd) of 77 ± 6 nM for aptamerT4 and 126 ± 34 nM for aptamerT1, which are similar to values determined by other techniques like surface plasmon resonance and capillary electrophoresis [16].

- Interface control: In solid-state pores, the aptamer can be tethered to the wall, so only in the target-bound state does the current blockade signature change significantly, improving specificity [17]. When this CEA-specific aptamer functionalized glass nanopore system was tested in human serum samples, nanopore-based quantification matched the ELISA results, indicating strong translational potential.
- Nanopipette and interface functionalization with aptamers for localized sensing: Aptamer-functionalized nanopipettes have recently emerged as powerful tools for localized, single-cell and subcellular sensing. By decorating the nanopipette tip with aptamers, the interface gains high molecular specificity, enabling selective recognition directly at the site of interest. The confined geometry of the nanopipette allows precise spatial control, while surface functionalization stabilizes the sensing interface and enhances signal-to-noise [14]. In another approach, the dynamic behavior of aptamer structures upon dopamine binding leads to the rearrangement of surface charge within the nanopore, resulting in measurable changes in ionic current. To assess sensor performance in real time, researchers have designed a fluidic platform to characterize the temporal dynamics of nanopipette sensors. Together, these advances make real-time, minimally invasive biochemical detection possible at unprecedented spatial resolution [19,21].
- Carrier-based scaffolds (DNA origami, λ-DNA) decorated with multiple aptamers to boost detection yield.

Carrier-based scaffolds such as DNA origami and λ-DNA enhance nanosensor performance by presenting multiple spatially organized aptamers, which increases effective affinity through multivalent (avidity-based) binding, boosts target capture rates, and prolongs binding lifetimes at nanopores or nanoelectrodes. Precise nanoscale positioning on DNA origami improves cooperative interactions, while long λ-DNA provides high-density decoration for enhanced encounter probability. This multivalency significantly improves signal strength, detection yield, and single-molecule capture efficiency in modern nano sensing platforms [22,23].

## 4. Recent Advancements of Aptamer-Based Nanopore Sensing

Recent applications of aptamer nanopore sensing have expanded significantly, moving beyond simple proof-of-concept studies into complex real-world diagnostics, environmental monitoring, and food safety. This technology combines the high specificity of aptamers (single-stranded DNA/RNA “chemical antibodies”) with the single-molecule sensitivity of nanopores (nanoscale holes that measure changes in ionic current).

### 4.1. Medical Diagnostics and Biomarker Detection

The most active area of research is in the rapid, label-free detection of disease biomarkers. Recent work has focused on detecting low-abundance biomarkers in complex biological fluids like blood or serum without extensive purification.

Cancer Biomarkers:

Recent sensors have been developed for lung cancer biomarkers (e.g., specific proteins or circulating tumor DNA). The aptamer acts as a gatekeeper; when it binds to the cancer marker, the complex is too large to pass through the pore or changes the “dwell time” (the time it takes to pass), creating a unique electrical signature. Nanopore sensors functionalized with anti-PSA aptamers have achieved detection limits in the picomolar range, far more sensitive than traditional ELISA tests.

Recently researchers have developed a specific DNA aptamer-based nanopore system to detect actinomycin-D, a potent anti-cancer drug. This sensor achieves high sensitivity, with a detection limit (LOD) of 1.20 nM, and is sensitive in complex human serum samples [24].

In recent review articles, a plethora of applications including cancer, metabolic disorders, and cardiovascular diseases have been discussed using aptamer–nanopore technology. This technology also facilitates early detection, multiplex analysis and translational diagnostics [25].

Infectious Diseases (Pathogens):

During the SARS-CoV2 pandemic, aptamer–nanopore systems were adapted to detect the spike protein of the virus. These sensors can distinguish between the original strain and variants (like Omicron) based on subtle differences in the binding signal [26,27].

Bacterial Pathogens: Systems have been designed to detect whole bacteria (like E. coli or Salmonella) or their specific toxins.

In a recent study, scientists have designed an aptamer self-assembly sensing strategy based on hydrophobic nanopores to identify the bacterial types in mixed samples. The proposed sensor exhibits a broad dynamic detection range for *Escherichia coli* and *S. aureus*. This electrochemical sensor showed high recognition sensitivity for low-concentration bacteria [28].

Neurodegenerative Diseases:

The current revival of aptamer technologies, combined with rapid progress in neuroscience, offers a powerful opportunity to deepen our understanding of core brain functions and dysfunctions. Structure-switching aptamers—defined by their high selectivity and target-induced conformational changes—are increasingly recognized as excellent recognition units for chemical biosensors. Yet, it remains crucial to evaluate each aptamer sequence under conditions that closely mimic the intended biological environment to ensure reliable and reproducible performance [29]. To detect dopamine, serotonin-like neurotransmitters, structure-switching aptamers are promising candidates for highly selective, label-free detection in complex biological environments [30]. The authors showcased aptamer-integrated FETs and nanopipettes that overcome Debye length limitations and achieve real-time, ultrasensitive, localized detection of dopamine and serotonin. Aptamer-coated FETs and nanopipettes showed high sensitivity and specificity in serum, CSF and undiluted biological media. Aptamers confined inside nanopipettes allow sub-cellular and synaptic-scale chemical detection with minimal tissue damage.

Aptamer-functionalized nanopore sensors combine the single-molecule sensitivity and label-free detection of nanopores with the high specificity and tunability of aptamers to detect disease-relevant protein biomarkers (e.g., misfolded or aggregated proteins) involved in neurodegeneration. This approach has already enabled detection of pathological aggregates such as oligomeric α-Synuclein in patient cerebrospinal fluid with minimal sample processing. Aptamers can also discriminate between different fibrillar “polymorphs” of the same protein—for example, different structural forms of α-Syn—potentially enabling differential diagnosis between related disorders. Due to the ability of nanopores to record individual binding or translocation events, this technology could support early diagnosis, real-time monitoring, and structural characterization of misfolded proteins in neurodegenerative diseases [21,25].

**Figure 3:**
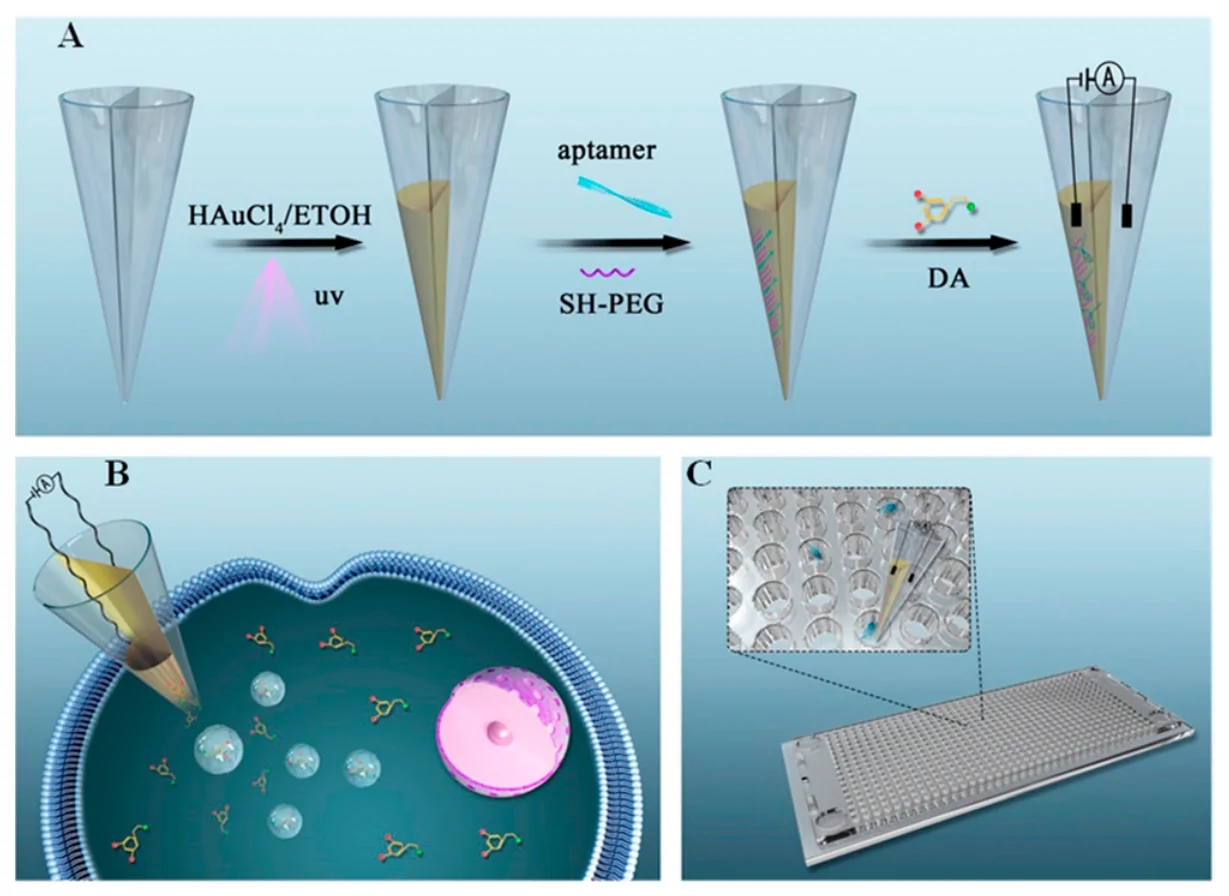
Schematic illustration of the dual-nanopore biosensor for dopamine detection based on various modes of DNA aptamer-mediated recognition. (**A**) Sequential steps of dual-nanopore biosensor for dopamine detection; (**B**) Dopamine detection at a single cell resolution by dual-nanopore sensor; (**C**) Detection of single-cell dopamine secretion on a microwell array by dual-nanopore sensor. (Reprinted with permission from ref. [29]. Copyright 2022, American Chemical Society).

Nanopore can also detect the difference between a single monomer (healthy) and an oligomer (toxic clump) based on the size of the current blockade (Figure 3).

### 4.2. Environmental Monitoring

Recent applications of aptamer–nanopore sensing have moved “out of the lab” to address field monitoring of pollutants, often focusing on heavy metals and small molecules that are difficult to detect with traditional methods like antibody-based systems. As of now, heavy-metal detection with aptamers (so-called “aptasensors”) has relied on optical or electrochemical readouts; the concept of combining aptamers with nanopores has started to gain attention to achieve single-ion sensitivity and “ultra-low” detection limits.

A recent report described a solid-state nanopore sensor functionalized with metal-ion-specific aptamers, demonstrating the detection of ions such as Hg^2+^, Pb^2+^, and Ag^+^ with high sensitivity—offering a compact, potentially portable platform for water-quality monitoring [31].

Specialized aptamers (like T-rich sequences for mercury) undergo a conformational change (e.g., forming a hairpin) when they bind the metal ions like mercury (Hg^2+^) and lead (Pb^2+^). This shape change alters the current flow through the nanopore, allowing for rapid quantification of water contamination. The integration of aptamers helps to overcome major challenges associated with heavy-metal sensing (small size, single binding site) by providing high specificity and selective binding, even in complex matrices like biological or environmental samples [32]. In a study, scientists studied the intrinsic mechanism of structure and charge-density distribution of aptamer-based resistive pulse sensors (RPSs). They opted for two metal-binding DNA aptamers (mercury and lead) to create a sensor that is efficient in a wide range of electrolytes, akin to river-to-sea water conditions [33].

There are various applications of nanopore sensing to detect pharmaceutical contaminants in the environment, like industrial waste, sewage water and wastewater systems. Sensors are now being used to detect antibiotics (e.g., tetracycline and kanamycin) in wastewater. This is crucial for tracking antimicrobial resistance in the environment.

### 4.3. Food Safety and Agriculture

**Figure 4:**
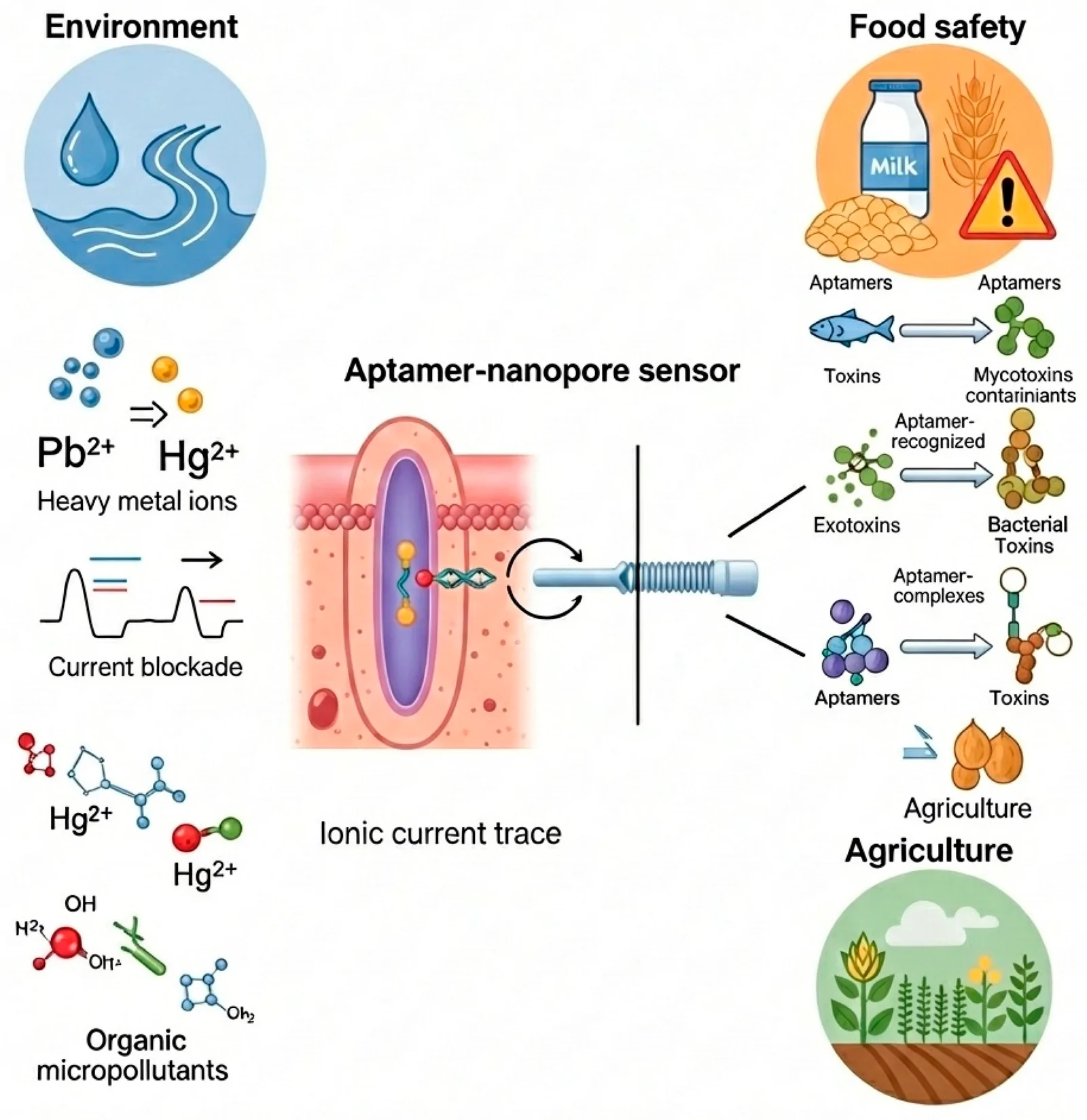
Aptamer–nanopore sensing applications in the environmental field (heavy metal detection and micropollutant detection), food safety applications (toxin and food contaminants) and the agriculture industry (pesticide, etc).

In the food industry, there is urgent demand for rapid, sensitive and portable detection tools that can identify contaminants and spoilage before the food enters the market. Aptamers are attractive candidates because they can be selected against many food-related pathogens like bacteria, viruses, etc., and nanopore-based platforms add another layer by enabling label-free, single-molecule settings and compact device designs. Thus, aptamer-based nanopore sensors align well with the need for portable food-safety testing (Figure 4).
**Nanopore Type****Aptamer Integration Strategy****Detection Limit****Strengths/Applications****Limitations****References**Biological nanopore, alpha hemolysin Aptamer tethering via DNA adapter, host- guest displacement assays, direct protein sensing, OTA sensing. Thrombin in the nM range; NP ~10 pM; OTA 1.697 pmol/L. Strong single-molecule readout, label-free, well-established platform, good for modular assay design and kinetic analysis. Requires fragile lipid bilayers, can be sensitive to matrix effects and pore stability issues. [34]Biological nanopore, MspANoncovalent docking of structure-switching aptamers to monitor conformational changes in dopamine, serotonin, and theophylline aptamers.Dopamine sensor with femtomolar range of detection. Excellent for conformational readout, real-time dynamics, and kinetic analysis of aptamer folding.Signal interpretation can be complex. [35]Quartz nanopipette-based solid-state nanoporeThiol-modified lysozyme-binding aptamers conjugated to 5 nm AuNPs. Selective single-molecule detection upto 50 nM lysozyme even in complex matrix.Good signal amplification, multiplexing, and improved capture from complex samples. Detection relied on strong aptamer–target affinity and relatively high analyte concentrations near the aptamer Kd range; additionally, small proteins still exhibited fast translocation and low intrinsic signal without the AuNP carrier.[36]Solid-state glass/quartz nanoporeThiolated aptamers immobilized on inner wall for CEA sensing and serum testing.CEA detection at 0.3 ng/µL. Mechanically robust, chemically modifiable, suitable for serum analysis and single-molecule event analysis.Surface fouling, nonspecific adsorption, and drift in pore properties can affect reproducibility. [37]Solid-state interface nanoporeDynamically tunable interface nanopore with amino-acid-specific aptamers for peptide recognition.Peptide discrimination at single-molecule resolution. Tunable pore geometry, can discriminate very similar peptide sequences, promising for proteomicsTranslocation control and nanoscale precision remain bottlenecks.[19]Nanopipette/interface nanoporeAptamer-functionalized nanopipettes for localized sensing and ion current rectification. Picomolar detection for serotonin sensing.Very useful for single-cell, subcellular, and minimally invasive measurements. Signal stability and calibration in complex media remain challenging. [38]Solid-state nanopores for metal ionsMetal-ion-specific aptamers for mercury (Hg), lead(Pb), silver (Ag) functionalized to develop Resistive Pulse Sensors (RPSs).Single particle resolution.Works in wide electrolyte ranges; suitable for environmental monitoring and water quality.Single-ion/small-analyte sensing is vulnerable to interference and nonspecific effects.[33]Solid-state hydrophobic nanoporesAptamer-anchored hydrophobic nanopores for bacteria discrimination and dual-mode readout.Broad dynamic detection range. Detection limits for *E. coli* and *S. aureus* are as low as 7.99 cfu/mL and 5.76 cfu/mL, respectively. Dual-mode detection, useful for complex samples and pathogen detection.Complex device architecture and assay workflow. [28]


Mycotoxins like Ochratoxin A (OTA) and Aflatoxin B1 are toxic compounds produced by mold on grains and coffee. Recently, nanopore-based sensors use a “competitive binding” assay: the toxin in the food sample competes with a DNA probe to bind the aptamer, changing the frequency of blockage events in the nanopore [39].

Recent studies have demonstrated the detection of organophosphorus pesticides (like acetamiprid) at trace amounts in sub-nanomolar concentrations. Researchers have introduced a dual signal nanopore biosensor that simultaneously measures ionic current and fluorescence to detect acetamiprid (ACE) residues with high sensitivity and specificity. By using a FAM-labeled aptamer–cDNA duplex grafted onto conical nanopores, ACE binding triggers structural changes that convert the sensor from a “closed/on” to an “open/off” state, enabling robust detection down to almost 0.12 ng/mL. The platform performs reliably in complex matrices from medicine–food homology products and shows excellent selectivity, stability, and agreement with conventional LC-MS technique. The aptamer binding prevents the pesticide from inhibiting downstream reactions or simply blocks the pore in a characteristic way [40]. The dual-mode nanopore design provides a simple, accurate, and highly sensitive approach for pesticide residue analysis, with strong potential for broader aptamer-based sensing applications.

Allergen Detection: New applications of sensors include detecting various food allergens like peanut (Ara h 1) or wheat (gluten) proteins in processed foods to ensure safety for allergic consumers [41]. Aptamer–nanopore systems offer a powerful platform for detecting such kind of food allergens like gluten peptides and other protein-based allergens. Aptamers provide high molecular recognition specificity, enabling them to selectively bind allergenic proteins even within complex food matrices [42]. When an allergen binds to its specific aptamer, which is tethered inside or at the entrance of a nanopore, binding induces a change in conformation or charge that modulates ionic current, producing a unique electrical signature for detection. This nanoscale confinement enhances sensitivity because binding events significantly alter ion flow, allowing label-free, real-time quantification at very low concentrations. Nanopores can also be engineered with multiple aptamers to detect several allergens simultaneously. Overall, aptamer–nanopore sensors offer fast, portable, and highly sensitive allergen monitoring, improving food safety and supporting rapid on-site testing [43].

## 5. Machine Learning Integration in Aptamer–Nanopore Sensing Signal Readouts and Analytical Capabilities

Machine learning (ML) significantly enhances the analytical performance of aptamer–nanopore sensors by improving how ionic current signals are interpreted and enabling more accurate, automated detection. ML enables aptamer–nanopore sensors to convert complex ionic current traces into quantitative, real-time readouts of target binding and kinetics with improved sensitivity and specificity. By learning characteristic current blockade patterns associated with aptamer conformations or aptamer–target complexes, ML classifiers (e.g., SVMs, HMMs, CNNs, and deep networks) can automatically detect and label translocation or docking events, even when they are highly noisy or short-lived. Firstly, ML algorithms can denoise and deconvolute complex current traces, making it easier to distinguish true binding events from background fluctuations in real time [44]. Secondly, pattern-recognition models—such as convolutional neural networks (CNNs) and recurrent neural networks (RNNs)—can classify subtle current blockade signatures to identify specific allergens or distinguish between closely related targets. Thirdly, ML enables quantitative prediction of analyte concentration by learning correlations between signal features (duration, amplitude, dwell time, and frequency) and analyte levels. Advanced approaches such as multivoltage “voltage-matrix” profiling combined with ML further expand analytical capabilities by exploiting voltage-dependent current signatures to differentiate targets or binding states in complex mixtures, including aptamer–biomarker complexes. Finally, ML supports multiplexed sensing by differentiating multiple aptamer–target interactions within the same nanopore, enhancing throughput and reducing false positives.

The specific features that can capture aptamer conformational changes are those that describe discrete current levels and their dynamics, rather than only single-event amplitude or duration. Key engineered features include the mean residual current at each blockade substate (multiple $I/I_{0}$ levels), their variance, and transition probabilities between these levels, which reflect distinct conformations and switching pathways. Dwell-time distributions for each substate, including multi-exponential components and state lifetimes, are especially informative for kinetic differences between unbound, partially bound, and fully bound aptamer conformations. Additional useful features are event-level statistics such as blockade depth, rise/fall times, and inter-event intervals, which encode changes in aptamer shape geometry, charge distribution, and flexibility. For nanopipette and wall-grafted aptamers, effective surface charge density, rectification ratio, and conductance–voltage curves provide indirect but highly sensitive descriptors of aptamer expansion–contraction and layer permeability changes [13,45]. In deep learning pipelines, raw or minimally processed current time series, optionally segmented into windows around conformational transitions, allow convolutional and recurrent networks to learn higher-order temporal patterns that correlate with hidden structural rearrangements [45]. Combining these level-resolved electrical features with biophysical information (e.g., MD- or FEM-informed charge-density changes) yields the most discriminative feature sets for machine learning models that classify aptamer conformational states in nanopore sensors. Overall, integrating ML into aptamer–nanopore systems transforms raw ionic signals into accurate, automated, and high-resolution biochemical information, making these sensors more robust for real-world biosensing applications.

In a study by Koch et al., they attempted to develop machine learning-driven nanopore sensing for quantitative, label-free detection of miRNAs. They exploited the moving standard deviation model (MSD), spectral entropy model (SE), and convolutional neural network (CNN) model for classifying delayed and non-delayed events. The concentration-response curves were sigmoidal for all three methods, and the nanopore readout agreed well with RT-qPCR validation. Based on input image analysis and manual thresholding, all three models can distinguish signal cases, but the CNN showed better sensitivity and higher robustness. The study underscores how crucial signal interpretation is in nanopore sensing and offers a useful comparison framework for binary event classification [46].

A key emerging challenge in aptamer–nanopore sensing is that aptamer binding often produces multilevel ionic blockade patterns and dynamic conformational switching rather than simple binary open/closed events. These complex signatures arise from transient intermediate states, partial folding/unfolding, target-induced structural rearrangements, and variable docking geometries within the nanopore. Consequently, apart from conventional amplitude-duration descriptors, feature engineering also includes state-transition probabilities, dwell-time distributions, fluctuation spectra, temporal correlations, and voltage-dependent blockade trajectories. Event labeling also becomes significantly more challenging because blockade states may overlap and represent hidden conformational intermediates that are not evident in the experimental setup. This necessitates probabilistic or weakly supervised labeling approaches, including Hidden Markov Models (HMMs) and self-supervised learning strategies. Furthermore, model design should also consider the sequential and hierarchical nature of nanopore signals; therefore, recurrent neural networks, temporal convolutional networks, transformers, and hybrid physics-led ML architectures are particularly attractive for capturing time-dependent conformational dynamics. Importantly, integrating biophysical knowledge of aptamer folding, electrostatic confinement, and nanopore transport into ML pipelines can improve interpretability and robustness. So, bridging a strong connection between machine learning and the underlying physics and chemistry plays a pivotal role in aptamer–nanopore sensing.

## 6. Challenges and Bottlenecks

Aptamer–nanopore sensing technology holds significant promise—offering advantages such as single-molecule sensitivity, high specificity, and potential for multiplexed analysis. However, several inherent limitations still restrict its broader applicability.

Limited specificity and interference in complex samples

Aptamer–nanopore platforms often struggle with distinguishing closely related analytes and suppressing background events, especially in serum or other complex matrices where non-specific adsorption and co-translocating species interfere with current signatures. Weak or suboptimal aptamer affinity and off-target binding further reduce selectivity and can produce overlapping blockade patterns. These issues complicate quantitative analysis at low concentrations and hinder reliable multiplexing. Strategies such as carrier constructs (aptamer-modified DNA or nanoparticles) improve specificity but add extra complexity and may introduce new artifacts [31].

Pore stability, biofouling, and device robustness

Biological nanopores depend on fragile lipid bilayers, which suffer from limited lifetime, instability under varying voltages or ionic strengths, and challenges in controlled insertion and long-term operation. Solid-state nanopores provide better mechanical robustness but face severe problems with non-specific adsorption, pore clogging, and drift in effective pore geometry and surface charge over time. Biofouling caused by proteins and other biomolecules can disrupt baseline currents and hinder aptamer accessibility, thereby compromising sensitivity and reproducibility [21]. These material-related challenges remain a significant obstacle to the translation of aptamer–nanopore devices into point-of-care measurements [6,47].

Signal-to-noise, temporal resolution, and data analysis load

Single-molecule events can be extremely fast and of small amplitude, requiring high-bandwidth, low-noise electronics and careful filtering to resolve aptamer conformational transitions and binding states. Thermal noise, flicker noise (1/f), and instrumental artifacts can mask subtle conformational signatures, while aggressive filtering risks distorting dwell times and amplitudes. Extracting kinetic and structural information demands sophisticated event detection, feature extraction, and often machine learning pipelines, which are computationally intensive and sensitive to parameter choices and training data bias. This bottleneck complicates standardization across various laboratories and hinders adaptation of the technology outside specialist groups [7].

Aptamer selection, structural constraints, and generalizability

SELEX-derived nucleotide aptamers may not possess the optimal folding pathways, mechanical properties, or electrostatic profiles needed to generate robust, distinguishable nanopore signals upon target binding [28,33]. Strong secondary structures (e.g., stable G-quadruplexes) can resist nanopore pulling or produce complex, heterogeneous blockade patterns that are difficult to interpret quantitatively. Additionally, aptamer performance often degrades in biological complex matrix conditions due to nuclease degradation (especially RNA aptamers), ionic-strength changes, and competition from endogenous ligands. These factors limit the transferability of a given aptamer–nanopore assay across targets, sample types, and operating conditions, making platform-wide standardization challenging. In solid-state nanopores, surface chemistry of modified aptamers can lead to nonspecific adsorption, which can clutter the signal unless carefully specified [9].

## 7. Future Directions and Conclusions

Future development in aptamer–nanopore sensing for biological applications is moving toward clinically relevant precision diagnostics and real-time monitoring of several biomarkers at ultra-low concentrations. Advances in various nanopore materials, aptamer engineering, and AI-assisted analysis are expected to make these platforms more robust, selective, and deployable in realistic biological environments [6]. Even in situ evolution of aptamers guided by nanopore signatures offers great promise.

In point-of-care and personalized diagnostics, aptamer-functionalized nanopores could be incorporated into portable or wearable devices for the rapid detection of disease biomarkers, pathogens, and drug molecules directly from blood, saliva, or urine samples. By combining multiplex aptamer panels with high-throughput nanopore arrays, it may become possible to measure proteins, nucleic acids, and metabolites in parallel, supporting earlier diagnosis and more effective disease monitoring.

A second important direction is neurobiology and cell biology, where nanopore or nanopipette platforms equipped with aptamer probes could be used to detect neurotransmitters and signaling molecules at or near the single-cell level. Such systems may allow localized measurement of molecules such as dopamine, serotonin, and neuropeptides, thereby improving mechanistic studies of neurological disorders and pharmacological responses, as noted by Stuber and Nakatsuka et al [35].

A third future direction is the use of aptamer–nanopore systems to also study biomolecular structure and conformational dynamics. By detecting subtle minor structural changes in proteins, peptides, or nucleic acids at the single-molecule level, these sensors could be valuable for epigenetic analysis, protein misfolding research, and drug–target interaction mapping in pharmaceutical development.

Nanopore biosensing to bridge fundamental research and real-world diagnostics, paving the way for rapid, sensitive, and accessible health monitoring tools in precision medicine. Integration with microfluidics, advanced nanofabrication, and machine learning is expected to yield automated lab-on-chip platforms that perform sample preparation, sensing, and data interpretation in a unified workflow. Such systems aim to overcome current bottlenecks of stability, biofouling, and data overload, enabling scalable, high-throughput aptamer–nanopore assays for routine biological and clinical specimens.

## Data Availability

No new data were created or analyzed in this study. Data sharing is not applicable to this article.
